# Infant deaths from respiratory syncytial virus in Lusaka, Zambia from the ZPRIME study: a 3-year, systematic, post-mortem surveillance project

**DOI:** 10.1016/S2214-109X(21)00518-0

**Published:** 2022-01-18

**Authors:** Christopher J Gill, Lawrence Mwananyanda, William B MacLeod, Geoffrey Kwenda, Rachel Pieciak, Zachariah Mupila, Caitriona Murphy, Chilufya Chikoti, Leah Forman, Flora Berklein, Rotem Lapidot, Charles Chimoga, Benard Ngoma, Anna Larson, James Lungu, Ruth Nakazwe, Diana Nzara, Lillian Pemba, Baron Yankonde, Angel Chirwa, Magda Mwale, Donald M Thea

**Affiliations:** a Department of Global Health, Boston University School of Public Health, Boston, MA, USA; b Biostatistics and Epidemiology Data Analytics Center, Boston University School of Public Health, Boston, MA, USA; c Department of Biomedical Sciences, School of Health Sciences, University of Zambia, Lusaka, Zambia; d Right to Care Zambia, Lusaka, Zambia; e Department of Pediatrics, Boston University School of Medicine, Boston, MA, USA; f Department of Psychiatry, University Teaching Hospital, University of Zambia School of Medicine, Lusaka, Zambia

## Abstract

**Background:**

Respiratory syncytial virus (RSV) is the leading cause of acute lower respiratory tract infections and a key driver of childhood mortality. Previous RSV burden of disease estimates used hospital-based surveillance data and modelled, rather than directly measured, community deaths. Given this uncertainty, we conducted a 3-year post-mortem prevalence study among young infants at a busy morgue in Lusaka, Zambia—the Zambia Pertussis RSV Infant Mortality Estimation (ZPRIME) study.

**Methods:**

Infants were eligible for inclusion if they were aged between 4 days and less than 6 months and were enrolled within 48 h of death. Enrolment occurred mainly at the University Teaching Hospital of the University of Zambia Medical School (Lusaka, Zambia), the largest teaching hospital in Zambia. We extracted demographic and clinical data from medical charts and official death certificates, and we conducted verbal autopsies with the guardian or next of kin. RSV was identified using reverse transcriptase quantitative PCR and stratified by age, time of year, and setting (community *vs* facility deaths). By combining the PCR prevalence data with syndromic presentation, we estimated the proportion of all infant deaths that were due to RSV.

**Findings:**

The ZPRIME study ran from Aug 31, 2017, to Aug 31, 2020, except for from April 1 to May 6, 2020, during which data were not collected due to restrictions on human research at this time (linked to COVID-19). We enrolled 2286 deceased infants, representing 79% of total infant deaths in Lusaka. RSV was detected in 162 (7%) of 2286 deceased infants. RSV was detected in 102 (9%) of 1176 community deaths, compared with 10 (4%) of 236 early facility deaths (<48 h from admission) and 36 (5%) of 737 late facility deaths (≥48 h from admission). RSV deaths were concentrated in infants younger than 3 months (116 [72%] of 162 infants), and were clustered in the first half of each year and in the poorest and most densely populated Lusaka townships. RSV caused at least 2·8% (95% CI 1·0–4·6) of all infant deaths and 4·7% (1·3–8·1) of community deaths.

**Interpretation:**

RSV was a major seasonal cause of overall infant mortality, particularly among infants younger than 3 months of age. Because most RSV deaths occurred in the community and would have been missed through hospital-based surveillance, the global burden of fatal RSV has probably been underestimated.

**Funding:**

Bill & Melinda Gates Foundation.

## Introduction

Acute lower respiratory tract infections (ALRI) are the leading cause of childhood deaths worldwide.^1^, ^2^ The Pneumonia Etiology Research in Child Health (PERCH) study identified respiratory syncytial virus (RSV) as the leading cause of severe ALRI, particularly among young infants (<6 months). With an aetiological fraction of 31%, RSV caused three-times more ALRI cases than did the next highest named pathogen.^3^

Proposed strategies for reducing RSV deaths include population-level preventative strategies using maternal RSV vaccines or infant monoclonal antibodies, of which several are in late-phase development. Trials have shown the efficacy of such strategies for preventing severe RSV disease and hospitalisations, even among full-term infants.^4^, ^5^, ^6^

Assuming these products are ultimately licensed, introducing them at a global scale is a massive undertaking that must be justified by a sufficient burden of disease. A meta-analysis estimated up to 120 000 infant deaths from RSV per year, of which 99% occur in low-income and middle-income countries.^7^ However, the estimated burden of community RSV deaths was based on modelling, not direct measurements of RSV prevalence among community deaths. Depending on the size of this unmeasured fraction, we could be underestimating global RSV deaths.

**Research in context**

**Evidence before this study**

In the Pneumonia Etiology Research in Child Health study, respiratory syncytial virus (RSV) was identified as a leading cause of pneumonia, with a population attributable fraction three times larger than that of the next named pathogen. Less clear is the global burden of fatal disease due to RSV. Meta-analyses, based largely on data from hospital settings, estimate around 120 000 child deaths due to RSV each year, 99% of which are concentrated in low-income and middle-income settings. A limitation of this approach is that the data on community deaths were not measured directly, but rather modelled based on a series of assumptions about the size of this unmeasured fraction.

**Added value of this study**

This study used systematic surveillance to directly measure, rather than estimate, the previously unmeasured proportion of infants who died from RSV outside hospital settings, and hence outside the usual surveillance systems. The study was highly representative, capturing nearly 80% of all infant deaths that occurred in Lusaka, Zambia over a 3-year period. The study included facility and community deaths, allowing us to directly observe the contribution from each category. Our key finding was that RSV is present in 7–9% of deceased infants younger than 6 months. We also note that RSV deaths are concentrated among infants younger than 3 months and that nosocomial RSV is common. Importantly, around two-thirds of RSV deaths occurred in the community and would have been missed through hospital-based surveillance. The added value of our study is two-fold—first, we directly measured this previously unmeasured fraction of RSV deaths, and second, we showed that this previously unmeasured fraction comprised most RSV deaths.

**Implications of all the available evidence**

We found that RSV is a major cause of infant mortality. The largest concentration of RSV deaths was detected among infants who never reached medical care, and therefore would be systematically missed from previous burden of disease estimates. The fact that most RSV deaths occur outside facility settings suggests that general improvements to health-care delivery are unlikely to substantially reduce RSV deaths in infants. By contrast, preventative strategies, such as vaccines or monoclonal antibodies, could be highly impactful.

We addressed this concern by directly measuring facility and community RSV deaths through a systematic post-mortem surveillance project in Zambia. We addressed the following questions: what is the prevalence of RSV among deceased infants; how does this prevalence differ by setting (community *vs* facility) and age; and what is the proportion of all-cause infant mortality due to RSV?

## Methods

### Study overview and ethical oversight

The Zambia Pertussis RSV Infant Mortality Estimation (ZPRIME) study was a 3-year post-mortem surveillance study among young infants in Lusaka, Zambia. Post-mortem studies offer a direct measure of the fatal burden of an infectious disease and, when capturing a high proportion of deaths, are robust against selection biases.

ZPRIME's protocol was approved by an external scientific advisory committee with expertise in epidemiology, statistics, pathology, and RSV virology (see acknowledgments at the end of the paper). ZPRIME was approved by the ethical review boards at Boston University Medical Center and the University of Zambia. Written informed consent was obtained from the infants’ next of kin or guardian.

### Study setting and data collection

Infants were eligible for inclusion if they were aged between 4 days and less than 6 months and were enrolled within 48 h of death. Enrolment occurred mainly at the University Teaching Hospital (UTH) of the University of Zambia Medical School (Lusaka, Zambia), the largest teaching hospital in Zambia. More than 80% of all people who have died in Lusaka subsequently pass through UTH. We also enrolled infants at several level-one satellite facilities, akin to small community hospitals. These facilities had only basic capacity, which at the time of the study did not include supplemental oxygen or suction, which are essential supportive care for infants with severe RSV. These satellite sites issued very few death certificates (one to two per site per month) making it impractical to deploy a full-time team member at each site. Instead, we prioritised data collection at the UTH morgue, collecting detailed clinical and demographic information from each infant in what we refer to as long-form data.

For the satellite site deaths, we partnered with local mortuary staff to obtain informed consent, nasopharyngeal swabs, and document basic demographic data, including age, sex, and date of death. We refer to these as short-form data. The main limitations of short-form data are that they do not measure the time from admission to death and cannot distinguish early facility deaths (<48 h) from late facility deaths (≥48 h), and that they lack clinical information to adjudicate deaths as respiratory versus non-respiratory for population attributable fraction calculations. To assess the completeness of our surveillance, we compared our enrolled totals against Lusaka's official age-specific burial registries during the observation period.

### Clinical data extraction

For deaths at UTH, we extracted demographic and clinical data from medical charts and official death certificates. For hospital long-form deaths, we conducted a verbal autopsy with the guardian or next of kin. The verbal autopsy is a tool developed by WHO for assigning syndromic causes of death when a physical autopsy cannot be done. We used the abbreviated verbal autopsy tool validated by the Institute for Health Metrics and Evaluation.^8^ Resting on the recall of non-medical individuals, the verbal autopsy has restricted accuracy in assigning a syndromic cause of death. However, we restricted our use of verbal autopsy data to the simpler task of sorting deaths into respiratory versus non-respiratory categories.

### Biological sample collection, storage, and laboratory procedures

We sampled the posterior nasopharynx of infants using flocked-tipped nylon swabs (Copan Diagnostics; Murietta, CA, USA). Swabs were transported in 3 mL universal transport media at 2–8°C before storage at –80°C. Total nucleic acid was extracted using the NucliSens EASYMAG system (Marcy l’Etoile, France).^9^, ^10^ We used reverse transcriptase quantitative PCR to identify RSV following the protocol developed by the respiratory viruses branch at the US Centers for Disease Control and Prevention (CDC).^11^ We tested each sample for the constitutive human enzyme RNAseP to show adequate sampling and the absence of PCR inhibitors.

We ran PCR for 45 cycles and defined a positive test result as a reaction showing a logarithmic fluorescence curve reactive at a cycle threshold less than 40, with an RNAseP cycle threshold less than 40, and with positive and negative plate controls performing as expected. Although cycle threshold is a continuous variable that is essentially the inverse of the viral load, studies commonly apply a cutpoint for adjudicating PCR values, although the selection of these (eg, <30 *vs* <40) is arbitrary and hence controversial.^12^ We adopted the less than 40 threshold for two reasons. First, it was the cutpoint used for an earlier multiplex assay that used the same RSV primers and probes as the CDC assay.^13^ Second, most RSV epidemiological studies identify cases during acute illness, when the PCR signal intensity is probably at its peak. Here, we identified RSV postmortem, a point where the PCR signal might have declined, showing the need for a more sensitive cutpoint.

### Statistical analysis

We sorted infant deaths into three categories based on location and timing of death as follows: brought in dead (infants who died outside medical care, ie, community deaths), early facility deaths (infants who died <48 h after hospitalisation), and late facility deaths (infants who died ≥48 h after hospitalisation).

RSV is exclusively a respiratory disease. In the PERCH study, RSV was rarely detected among asymptomatic controls.^3^ Logically, if RSV participates in a fatal chain of events, RSV deaths should be concentrated among infants with an apparent respiratory syndrome. Therefore, in population attributable fraction calculations, we used the long-form clinical data to adjudicate each death as respiratory, non-respiratory, or uncertain, and we overlaid these categorisations against the RSV PCR results. Three study clinicians (CJG, DMT, and LM) independently reviewed each long-form death and assigned them to one of these categories. Discordant adjudications were discussed by the trio and either resolved as respiratory or non-respiratory, or remained uncertain if a consensus could not be achieved. The clinicians were masked to the PCR results throughout this process.

We did not conduct a formal power calculation around RSV. Rather, the power assumptions of the ZPRIME study were based on the predicted prevalence of pertussis, the other pathogen being studied. Because pertussis is far less common than RSV, we assumed that samples sizes needed to precisely measure pertussis would be sufficient for RSV. Our pertussis results are being prepared for publication separately.

Our analysis had three aims: aim 1 (proportion of RSV among all deaths) is a straightforward prevalence calculation, where the numerator is RSV-positive deaths, and the denominator is all infant deaths; aim 2 stratifies prevalence by community versus facility, and into early facility deaths versus late facility deaths; and aim 3 follows the approach of the Global Enteric Multicenter Study to estimate the population attributable fraction for deaths caused by RSV.^14^, ^15^

The first step in the population attributable fraction yields the risk ratio (RR) of exposure. In this study, because all infants were deceased, we modified the usual formula for RR of exposure from the familiar four-cell contingency table as described in the appendix (p 1).

The next step yields the population attributable fraction by adjusting RSV prevalence by the RR of exposure,

PR(A|Death)(1-1RRe)

where PR means proportion, A is the proportion of deceased infants who are RSV-positive, and RRe is the risk ratio of exposure.

In sensitivity analyses, we evaluated the effect on the population attributable fraction by assigning cases that remained uncertain after adjudication to either the respiratory or non-respiratory categories.

We used ArcGIS to map deaths using data from the Zambia Data Hub. We used SAS version 15.2 for all statistical analyses.

### Role of the funding source

The objectives of the ZPRIME study were agreed upon with our funder at the start of the project. Beyond that, the funder of the study had no role in data collection, data analysis, data interpretation, or writing of the report.

## Results

The ZPRIME study ran from Aug 31, 2017, and Aug 31, 2020. All human research in Zambia was suspended from April 1**,** to May 6, 2020, because of COVID-19, creating a surveillance gap. We addressed this issue by presenting RSV prevalence for all years combined and restricted to years with uninterrupted data.

**Figure 1:**
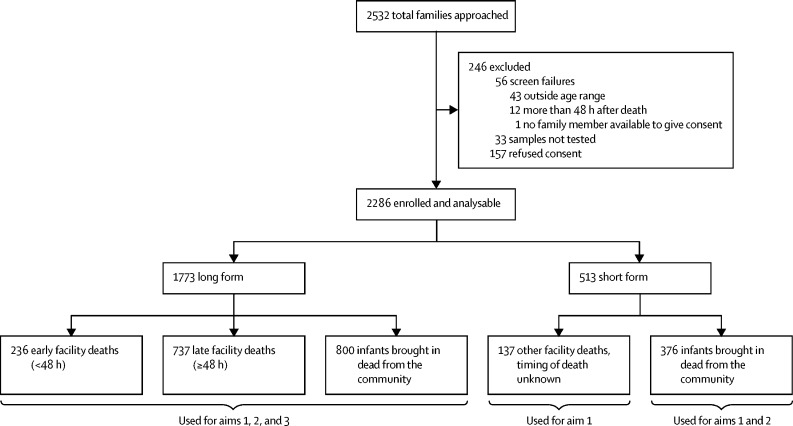
Study enrolment flow diagram Infants who died at sites other than UTH or the four satellite sites that could issue death certificates could contribute deaths outside our enrolment system. Most of our data were collected at UTH, yielding detailed long-form data. These data had sufficient detail to be used in all three study aims. By contrast, the limited short-form data, collected at satellite sites, contributed mainly to aim 1 and, to a partial extent, aim 2. The three aims were: aim 1 (proportion of RSV among all deaths) is a straightforward prevalence calculation, where the numerator is RSV-positive deaths, and the denominator is all infant deaths; aim 2 stratifies prevalence by community versus facility, and into early facility deaths versus late facility deaths; and aim 3 follows the approach of the Global Enteric Multicenter Study to estimate the population attributable fraction for deaths caused by RSV. UTH=University Teaching Hospital of the University of Zambia Medical School.

We enrolled 2286 deceased infants (figure 1). Comparing our totals against official burial registries, we enrolled 79% of all infants who died in Lusaka between the ages of 4 days and 6 months during the surveillance period. We collected long-form data on 1773 (78%) infants and short-form data on 513 (22%) infants. 1176 (51%) of 2286 infants were brought into hospital dead (community deaths), 737 (32%) were late facility deaths, and 236 (10%) were early facility deaths.

Participant demographics are summarised in table 1. All infants were of African ethnicity. Roughly half of participants were female. The median age at death was 28 days (IQR 9–86). Infants who were brought in dead were significantly older than those who died at a facility (60 days *vs* 17 days for early facility deaths and 10 days for late facility deaths; p<0·0001, Cochran–Mantel–Haenszel test). This finding could be explained by the higher proportion of late facility deaths complicated by common perinatal conditions (predominantly prematurity, low birthweight, or complications of labour and delivery).

**Table 1:** Demographics of deceased infants by location and timing of death

		**Infants brought in dead (community deaths; n=1176)**	**Early facility deaths (<48 h; n=236)**	**Late facility deaths (≥48 h; n=737)**	Facility deaths, time to death unknown*(n=137)	**All infants (n=2286)**
Sex						
	Female	526/1016 (52%)	101/236 (43%)	347/732 (47%)	60/119 (50%)	1034/2103 (49%)
	Male	490/1016 (48%)	135/236 (57%)	385/732 (53%)	59/119 (50%)	1069/2103 (51%)
	Missing	160 (14%)	0	5 (1%)	18 (13%)	183 (8%)
Age at death						
	Median age at death (IQR), days	60 (21–107)	17 (7–64)	10 (5–24)	28 (7–94)	28 (9–86)
	4–7 days	97 (8%)	67 (28%)	295 (40%)	36 (26%)	495 (22%)
	8–14 days	117 (10%)	40 (17%)	167 (23%)	15 (11%)	339 (15%)
	15–28 days	152 (13%)	34 (14%)	107 (15%)	18 (13%)	311 (14%)
	1–2 months	430 (37%)	51 (22%)	103 (14%)	33 (24%)	617 (27%)
	3–5 months	380 (32%)	44 (19%)	65 (9%)	35 (26%)	524 (23%)
Median days hospitalised before death (IQR)		NA	1 (0–1)	6 (4–11)	UN	5 (2–9)
Discharged home after birth		NA	175/175 (100%)	345/707 (49%)	UN	520/882 (59%)
HIV status of mother						
	HIV-positive	24/144 (17%)	54/235 (23%)	140/716 (20%)	UN	218/1095 (20%)
	HIV-negative	120/144 (83%)	181/235 (77%)	576/716 (80%)	UN	877/1095 (80%)
	Unknown	1032 (88%)	1 (<1%)	21 (3%)	137 (100%)	1095 (48%)
Uneventful birth history†		UN	215/227 (95%)	608/714 (85%)	UN	823/941 (87%)
Respiratory deaths*						
	Death adjudicated‡	800 (68%)	236 (100%)	737 (100%)	0	1773 (78%)
	Respiratory death	316/800 (40%)	94 (40%)	105 (14%)	UN	515/1773 (29%)

Data are n/N (%) or n (%), unless otherwise indicated. NA=not applicable. UN=unavailable.

* From satellite facilities; for these low volume recruitment sites, we collected minimal information that did not include time since admission to death and so could not be categorised as early or late facility deaths—similarly, because these deaths lacked clinical information needed for syndromic adjudication, they were not used for population attributable fraction estimates.

† Uneventful births exclude pre-term births, low birthweight, or complications during pregnancy or labour and delivery.

‡ Verbal autopsy data for syndromic adjudication of infants who were brought in dead were only collected for the 800 (68%) of 1176 infants enrolled at the University Teaching Hospital of the University of Zambia Medical School morgue, and not for the 37 (32%) of 1176 infants who were brought in dead enrolled at satellite sites; maternal HIV status was rarely known for the infants who were brought in dead.

Overall, 514 (29%) of 1772 infant deaths were adjudicated as respiratory deaths, but this proportion was far higher among infants who were brought in dead (316 [40%] of 800 infants) and early facility deaths (94 [40%] of 236 infants) than among late facility deaths (105 [14%] of 737 infants), again reflecting the concentration of deaths in this latter category related to prematurity or complications of pregnancy or labour and delivery, as these infants tended to be in the hospital for extended periods of time.

In summary, the demographic data depict distinct infant populations that differed in predicable and meaningful ways. The late facility deaths were heavily represented by infants who had been born at UTH, had multiple birth complications, and often did not survive long enough to enter the community. By contrast, infants who died in the community were well enough to have been discharged home. They were older with less complicated births, but were at increased risk of community exposure to circulating pathogens, such as RSV.

Nasopharyngeal samples were obtained at a median of 18·5 h since death among facility deaths and 13·8 h since death among community deaths (difference in means 4·6 h, 95% CI 3·7–5·6). This apparent paradox occurs because the deaths occurring overnight at the paediatric wards are not immediately transferred to the morgue, but rather brought over each morning. By contrast, infants who were brought in dead came directly to the morgue.

RNAseP was detected in all samples and the mean RNAseP cycle threshold values did not differ significantly between infants with and without RSV (mean value for RSV-positive infants 25·2 and for RSV-negative infants 26·3; difference in means –1·1, 95% CI –1·9 to –0·34).

Using the same groupings as in table 1, the demographics for RSV-positive infants are summarised in the appendix (pp 2–3). Overall, 162 (7%) of 2286 deceased infants tested positive for RSV at the cycle threshold of less than 40 (table 2). Among this group, the median cycle threshold value was 27·3. The mean RSV PCR cycle threshold values were very similar between the infants who were brought in dead versus those who died at a facility (mean cycle threshold 27·9 *vs* 29·7, respectively; difference in means –1·8, 95% CI –4·4 to 0·8). The distribution of cycle thresholds for all RSV-positive infants is summarised in the appendix (p 6). An additional six infants had detectable RSV at cycle threshold greater than 40, but they were not included in our analyses.

**Table 2:** Summary of deceased infants by RSV PCR test status, year, setting, and age category

	**Infants brought in dead**		**Early facility deaths**		**Late facility deaths**		**Facility deaths, timing unknown**		**All infants**	
	Number tested	Number RSV-positive	Number tested	Number RSV-positive	Number tested	Number RSV-positive	Number tested	Number RSV-positive	Number tested	Number RSV-positive
**August to December, 2017**										
Age 4 days to <3 months	49	0	16	0	64	0	0	0	129	0
Age 3 months to <6 months	22	0	7	0	7	0	1	0	37	0
All ages	71	0	23	0	71	0	1	0	166	0
**January to December, 2018**										
Age 4 days to <3 months	308	26 (8%)	54	2 (4%)	159	7 (4%)	82	4 (5%)	603	39 (6%)
Age 3 months to <6 months	159	14 (9%)	12	0	21	1 (5%)	24	8 (33%)	216	23 (11%)
All ages	467	40 (9%)	66	2 (3%)	180	8 (4%)	106	12 (11%)	819	62 (8%)
**January to December, 2019**										
Age 4 days to <3 months	314	44 (14%)	90	5 (6%)	289	20 (7%)	10	1 (10%)	703	70 (10%)
Age 3 months to <6 months	138	8 (6%)	21	3 (14%)	26	6 (23%)	7	1 (14%)	192	18 (9%)
All ages	452	52 (12%)	111	8 (7%)	315	26 (8%)	17	2 (12%)	895	88 (10%)
January to August, 2020*										
Age 4 days to <3 months	125	5 (4%)	32	0	160	2 (1%)	10	0	327	7 (2%)
Age 3 months to <6 months	61	5 (8%)	4	0	11	0	3	0	79	5 (6%)
All ages	186	10 (5%)	36	0	171	2 (1%)	13	0	406	12 (3%)
**All years**										
Age 4 days to <3 months	796	75 (9%)	192	7 (4%)	672	29 (4%)	102	5 (5%)	1762	116 (7%)
Age 3 months to <6 months	380	27 (7%)	44	3 (7%)	65	7 (11%)	35	9 (26%)	524	46 (9%)
All ages	1176	102 (9%)	236	10 (4%)	737	36 (5%)	137	14 (10%)	2286	162 (7%)

RSV=respiratory syncytial virus.

* Missing April and May, 2020, because of COVID-19 shutdown in Lusaka.

RSV was present among 102 (9%) of 1176 infants who were brought in dead, ten (4%) of 236 early facility deaths, and 36 (5%) of 737 late facility deaths. Among infants who died at a satellite facility, where time from admission to death was unknown, 14 (10%) of 137 infants were RSV-positive.

**Figure 2:**
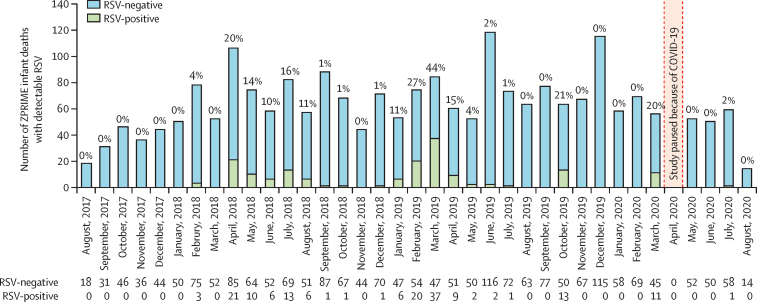
RSV deaths over time The histogram shows the numbers of infant deaths by month. The percentages at the top of each bar are the proportion of RSV-positive deaths out of total deaths in each month. RSV=respiratory syncytial virus.

Our findings probably underestimate the burden of RSV because of the gap in enrolments imposed by COVID-19 in April and May, 2020, just as the first cases of RSV were being detected by our study (figure 2); this is reflected in the numbers of RSV-positive deaths in 2018, 2019, and 2020 (62, 88, and 12 RSV deaths, respectively). When limiting our analysis to years with uninterrupted data, RSV was detected in 150 (9%) of 1704 infants, of which 102 (68%) of 150 infants were brought in dead. No cases of RSV were detected in 2017, plausibly because enrolment began on Aug 31, after the spring RSV season had passed. Among the 162 RSV-positive deaths, 102 (63%) were infants brought in dead, ten (6%) were early facility deaths, and 36 (22%) were late facility deaths.

116 (72%) of 162 RSV-positive infants were younger than 3 months (table 2). However, this finding must be interpreted considering the proportion of total deaths in that younger age range (1762 [77%] of 2286 infants). When adjusting for these proportions, younger infants aged between 4 days and less than 3 months appeared slightly less likely to die of RSV than older infants (RR 0·75, 95% CI 0·54–1·04; p=0·090). The mean RSV PCR cycle threshold values were somewhat higher for infants younger than 3 months (n=116) versus those aged 3 months and older (n=46; cycle threshold 30·3 *vs* 27·7; difference in means 2·6, 95% CI 0·05–5·10).

In most years, RSV transmission appeared to be concentrated in the first half of the year, typically peaking between March and April (figure 2). These seasonal trends probably explain the absence of cases detected from August to December, 2017, since in most years RSV transmission was concentrated in the spring months. The timing of the suspension of human research because of COVID-19 was unfortunate since RSV transmission had only just been detected in March, 2020, and few cases were detected after enrolments resumed in May, 2020. A cluster of cases in October, 2019, showed that RSV can transmit throughout the year. The geographical distribution of RSV deaths across Lusaka is shown in the appendix (p 7). RSV deaths were concentrated in the poorest and most densely populated Kanyama, Chipata, and Chawama townships. By contrast, few RSV deaths occurred in the wealthier Matero, Chilenje, and Chelstone townships.

Population attributable fraction estimates of deaths from ALRI that were adjudicated as being due to RSV, stratified by setting, are provided in table 3. The underlying data tables for these calculations are provided in the appendix (p 4). After adjudication, RSV was detected in 65 (13%) of 515 infants with a respiratory death versus 43 (4%) of 1121 infants with a non-respiratory condition (appendix p 1). RSV caused at least 2·8% of all-cause infant deaths (95% CI 1·0–4·6). However, this finding differed markedly by setting: 4·7% of deaths (1·3–8·1) among infants who were brought in dead were caused by RSV versus only 0·9% of late facility deaths (95% CI 0·0–3·2). In sensitivity analyses, these results remained similar when treating the uncertain deaths either as respiratory or non-respiratory deaths (appendix p 5).

**Table 3:** Population attributable fraction of deaths from acute lower respiratory tract infections that were adjudicated as being due to respiratory syncytial virus

	**Population attributable fraction**
All deaths	2·8 (1·0–4·6)
Infants brought in dead	4·7 (1·3–8·1)
Early facility deaths (<48 h from admission)	2·2 (0·0–6·1)
Late facility deaths (≥48 h from admission)	0·9 (0·0–3·2)

Data are % (95% CI).

Among the 36 RSV-positive infants with long-form data who died at UTH, 12 (33%) of 36 infants were born and died at the hospital without ever leaving and unambiguously represent nosocomial RSV. However, this result probably underestimates the nosocomial burden. The typical incubation period of RSV is 4–6 days, although shedding of virus among hospitalised patients has been documented for up to a week after diagnosis.^16^ The distribution of time from admission to death within this group is summarised in the appendix (p 8). Conservatively, if we assume that all ten deaths before day 5 were community acquired, occurring at the lower bound of the incubation period, but that some proportion of later deaths could be nosocomial, then the plausible range of nosocomial RSV deaths ranges from 12 (33%) of 36 infants to 23 (64%) of 36 infants. Viewed as a fraction of all infant RSV deaths, nosocomial deaths were uncommon, accounting for between 12 (7%) of 162 infants and 23 (14%) of 162 infants.

## Discussion

In this 3-year systematic post-mortem study, RSV was present in at least 7% of all deceased infants, and closer to 9% of infants when limiting our analysis to years with complete data. We also found that RSV deaths were concentrated among younger infants and that nosocomial transmission accounted for at least a third of late facility deaths. However, a clear majority of RSV deaths occurred in the community (ie, infants who were brought in dead). Such infants are not routinely tested for RSV postmortem and will not be captured in hospital-based surveillance studies. Consequently, these infants will be systematically omitted from global burden of RSV estimates. In our view, the most important contribution of ZPRIME was to directly measure this previously unmeasured fraction and show that it accounts for most RSV deaths.

Our results probably undercount the measured burden to some degree. First, we could not capture deaths as late sequelae of RSV after RSV is no longer detectable by PCR. Such deaths could include cases of bacterial pneumonia or other complications of severe illness, therefore deflating both the RSV prevalence and population attributable fraction estimates. Supporting this concern, a well-conducted longitudinal analysis of South African infants reported that ALRI rates were 3-times higher after RSV infection.^17^ Second, our study only included infants aged between 4 days and 6 months, but RSV deaths are not confined to the first 6 months of life. Third, our population attributable fraction calculations rest on post-hoc interpretation of medical information that was collected by others (for hospital deaths) or reported by others (for community deaths) through verbal autopsy. This fact could introduce some degree of random misclassification, biasing our results towards the null and underestimating the population attributable fraction.

The concentration of RSV deaths in the community has substantial implications for future policy investments. One approach would be to invest in strengthening community therapeutic capacity generally. The lower proportion of RSV-positive deaths among the early facility deaths and late facility deaths in our study suggests that supportive care might be life saving for infants who are able to reach a facility. However, this strategy would not necessarily improve access to care, which we have previously shown to be a common factor among community infant deaths,^18^ and would only be relevant to the minority of RSV deaths that occur at facilities.

Alternatively, investment could be made in population-level preventative interventions. The current systems that deliver tetanus toxoid to pregnant women and routine vaccines to infants could be leveraged to deliver maternal RSV vaccines or anti-RSV monoclonal antibodies.^19^, ^20^ This approach takes advantage of existing infrastructure and human capacity and is less dependent on care-seeking behaviour, timely access to transportation, or medical capacity at facilities. The high concentration of RSV among infants who were brought in dead from the community (rather than dying in facilities) strongly favours this approach.

RSV deaths were more common in infants younger than 3 months of age than those aged 3–6 months, accounting for around two-thirds of deaths. This finding aligns with our previous finding that younger infants were five times more likely to die of RSV than were older infants.^21^ Yet, this oversimplifies a more nuanced relationship between age and setting. In the community, RSV deaths were clustered among the youngest infants, whereas infants who died at facilities tended to be older. This apparent paradox might be explained by survival bias, case mix, and immunology. First, we draw attention to the far younger median ages at death for the late facility deaths versus community deaths. An infant cannot die of RSV unless surviving long enough to be exposed to RSV. Second, facility infant deaths were largely from neonatal complications from labour and delivery, pregnancy, or prematurity. These common risk factors for death might dilute the contribution from all other causes of mortality, including RSV. Finally, maternal antibodies could offer protection against RSV earlier in life.^22^, ^23^, ^24^

There were several strengths to our study. Previous analyses of community mortality in Argentina characterised the impact of RSV mortality in a densely populated urban community.^25^, ^26^ However, infant mortality rates in Argentina are far lower than those in Zambia, so our data provide insight into a different epidemiological context. Within that context, Lusaka is demographically similar to many other African cities with high population density, poverty, infant mortality rates, and HIV prevalence (around 12% according to 2020 UNAIDS estimates).^27^ We captured most infant deaths in Lusaka during the surveillance period, making our data highly representative. Limitations of our study are that the population attributable fraction calculations relied on the accuracy of clinical data, allowing for potential misclassification, and that all our data come from one city. Our current analysis does not address risk factors for RSV mortality, although these have been explored in detail in our other articles.^21^, ^28^

In conclusion, RSV was a major source of all-cause infant mortality in Lusaka. Since most infant RSV deaths occurred outside medical care and would have been missed in hospital-based surveillance systems, previous meta-analyses might have underestimated the global burden of fatal RSV. The concentration of RSV deaths in community settings and among infants younger than 3 months of age suggests that population-level RSV preventative strategies via maternal vaccines or monoclonal antibodies could have a high impact on reducing overall childhood mortality.

## Data sharing

We have pledged to share our data, including access to our biorepository, upon any reasonable request to the corresponding author. We have provided our datasets to several collaborating organisations.

## Declaration of interests

CJG and LM report grants from the US National Institutes of Health and from Merck, paid to Boston University. CJG also reports receiving fees for participation on data safety and monitoring boards for Takeda, Moderna, and CureVac, all outside the submitted work. LM reports receiving financial support from the Bill & Melinda Gates Foundation to attend meetings on respiratory syncytial virus. RL reports research awards from Pfizer, paid to her institution, and honoraria for participation in pneumococcal advisory boards and consulting fees from Pfizer, paid to her, outside the submitted work. AL reports employment with Manpower Professional Services, which was contracted by Merck to complete work for the Global Vaccines Public Policy & Partnerships team, and fees from Merck for consultancy services, outside the submitted work. All other authors declare no competing interests.
