# Supplementary material for: Infant deaths from respiratory syncytial virus in Lusaka, Zambia from the ZPRIME study: a 3-year, systematic, post-mortem surveillance project
Source: Lancet Glob Health. 2022 Jan 18;10(2):e269–77. doi: 10.1016/S2214-109X(21)00518-0 (PMC8789563; doi:10.1016/S2214-109X(21)00518-0)
Supplement: Supplementary appendix [file mmc1.pdf]

THE LANCET
Global Health

Supplementary appendix

This appendix formed part of the original submission and has been peer reviewed. We post it as supplied by the authors.

Supplement to: Gill CJ, Mwananyanda L, MacLeod WB, et al. Infant deaths from respiratory syncytial virus in Lusaka, Zambia from the ZPRIME study: a 3-year, systematic, post-mortem surveillance project. *Lancet Glob Health* 2022; **10:** e269–77.

SUPPLEMENTARY FILES

Supplementary Table S1: Modification of the 2x2 contingency table for calculation of the relative risk of exposure.

	Respiratory death	Non-respiratory death
RSV PCR(+)	a	b
RSV PCR(-)	c	d
	Where **RRe = R+ / R0**<br>This is defined as R+ is the proportion of RSV PCR(+) deaths<br>= (a+b) / (a+b+c+d),<br>and R0 is the RSV PCR(+) proportion among the non-respiratory deaths = b / (b+d).	

We use R+ and R0 since the cohort lacks 'controls' in the usual sense (cases ordinarily being those with the condition (i.e., deceased) and controls lacking the condition (i.e., alive)). Here, all infants were deceased.

Supplementary Table S2:
Demographic features of RSV positive deceased infants by setting

	Deaths in the community (Infants brought in dead)	Early facility deaths < 48 hours	Late facility deaths ≥ 48 hours	Facility deaths time to death unknown*	All RSV(+) infants
	(n=102)	(n=10)	(n=36)	(n=14)	(n=162)
RSV PCR positive % (n/N)	100% (102/102)	4.2% (10/236)	4.9% (36/737)	10.2% (14/137)	100% (162/162)
RSV PCR negative % (n/N)	0% (0/102)	0% (0/10)	0% (0/36)	0% (0/14)	0% (0/162)
RSV PCR unknown % (n/N)	0	0	0	0	0
Females % (n/N)	46.6% (41/88)	40.0% (4/10)	58.3% (21/36)	85.7% (12/14)	52.7% (78/148)
Males % (n/N)	53.4% (47/88)	60.0% (6/10)	41.7% (15/36)	14.3% (2/14)	47.3% (70/148)
Unknown Sex	14	0	0	0	14
Age at death					
Median age at death, days (IQR)	41.0 (23 - 101)	34.5 (11 - 120)	24.0 (10 - 66)	109.0 (62 - 124)	41.0 (18 - 107)
Mean age at death, days (sd)	60.7 (49.9)	54.7 (50.9)	45.1 (48.7)	97.5 (47.2)	60.0 (50.8)
4-7 days % (n/N)	5.9% (6/102)	20.0% (2/10)	22.2% (8/36)	7.1% (1/14)	10.5% (17/162)
8-14 days % (n/N)	8.8% (9/102)	20.0% (2/10)	16.7% (6/36)	0.0% (0/14)	10.5% (17/162)
15-28 days % (n/N)	18.6% (19/102)	10.0% (1/10)	13.9% (5/36)	0.0% (0/14)	15.4% (25/162)
1-2 months % (n/N)	40.2% (41/102)	20.0% (2/10)	27.8% (10/36)	28.6% (4/14)	35.2% (57/162)
3-5 months % (n/N)	26.5% (27/102)	30.0% (3/10)	19.4% (7/36)	64.3% (9/14)	28.4% (46/162)
Unknown age at death	0	0	0	0	0
Median days of hospitalization before death (IQR)	Not applicable	1.0 (0 - 2)	7.0 (4 - 15)	Unavailable	5.0 (3 - 12)
Discharged home after birth % (n/N)	Not applicable	100.0% (6/6)	64.7% (22/34)	Unavailable	70.0% (28/40)
Not discharged home after birth % (n/N)	Not applicable	0	51.2% (362/34)	Unavailable	30.0% (12/40)
Unknown discharge status	Not applicable	4	2	Unavailable	6
HIV Status of Mother					
Mother was HIV(+) % (n/N)	28.6% (2/7)	10.0% (1/10)	31.4% (11/35)	Unavailable	26.9% (14/52)
Mother was HIV(-) % (n/N)	71.4% (5/7)	90.0% (9/10)	80.4% (24/35)	Unavailable	73.1% (38/52)
Unknown Maternal HIV Status	95	0	1	Unavailable	96

Birth History					
Normal Birth** % (n/N)	Unavailable	88.9% (8/9)	85.3% (29/34)	Unavailable	86.0% (37/43)
Not Normal Birth % (n/N)	Unavailable	11.1% (1/9)	14.7% (5/34)	Unavailable	14.0% (6/43)
Unknown birth history	Unavailable	1	2	Unavailable	3
Respiratory Death*					
Death adjudicated % (n/N)	70.6% (72/102)	100.0% (10/10)	100.0% (36/36)	Unavailable	72.8% (148/148)
Deaths not adjudicated % (n/N)	0	0	0	Unavailable	0
Unable to be adjudicated	0	0	0	Unavailable	0
Respiratory Death % (n/N)	66.7% (48/72)	70.0% (7/10)	27.8% (10/36)	Unavailable	55.1% (65/118)
Not Respiratory Death % (n/N)	33.3% (24/72)	30.0% (3/10)	72.3% (26/36)	Unavailable	44.9% (53/118)
Unable to determine respiratory death	0	0	0	Unavailable	0

Supplementary Table S3:
Data used to generate Population Attributable Fraction (PAF) for deaths caused by RSV
ALRI = Acute Lower Respiratory Tract Infection
ALRI (I) = 'Unclear' if child had died with a respiratory vs. non-respiratory syndrome following the adjudication/reconciliation process.
These are the cases that were added to either the ALRI(+) or ALRI (-) groups for the sensitivity analyses on the PAF calculations (results of these analyses shown in **Table S4** below).

All Deaths

	ALRI(+)	ALRI(-)	ALRI(U)
RSV PCR (+)	65	43	10
RSV PCR (-)	450	1078	127
	515	1121	137

Cases adjudicated as ALRI (+) or (-)

	ALRI(+)	ALRI(-)
RSV PCR (+)	65	43
RSV PCR (-)	450	1078
	515	1121

Facility Deaths

	ALRI(+)	ALRI(-)	ALRI(U)
RSV PCR (+)	65	43	10
RSV PCR (-)	450	1078	127
	515	1121	137

Cases adjudicated as ALRI (+) or (-)

	ALRI(+)	ALRI(-)
RSV PCR (+)	65	43
RSV PCR (-)	450	1078
	515	1121

Community Deaths

	ALRI(+)	ALRI(-)	ALRI(U)
RSV PCR (+)	10	22	4
RSV PCR (-)	95	576	30
	105	598	34

Cases adjudicated as ALRI (+) or (-)

	ALRI(+)	ALRI(-)
RSV PCR (+)	10	22
RSV PCR (-)	95	576
	105	598

Cases adjudicated as ALRI (I) added to ALRI(+)

	ALRI(+)	ALRI(-)
RSV PCR (+)	75	43
RSV PCR (-)	577	1078
	652	1121

Cases adjudicated as ALRI (I) added to ALRI(-)

	ALRI(+)	ALRI(-)
RSV PCR (+)	65	53
RSV PCR (-)	450	1205
	515	1258

Cases adjudicated as ALRI (I) added to ALRI(+)

	ALRI(+)	ALRI(-)
RSV PCR (+)	75	43
RSV PCR (-)	577	1078
	652	1121

Cases adjudicated as ALRI (I) added to ALRI(-)

	ALRI(+)	ALRI(-)
RSV PCR (+)	65	53
RSV PCR (-)	450	1205
	515	1258

Cases adjudicated as ALRI (I) added to ALRI(+)

	ALRI(+)	ALRI(-)
RSV PCR (+)	14	22
RSV PCR (-)	125	576
	139	598

Cases adjudicated as ALRI (I) added to ALRI(-)

	ALRI(+)	ALRI(-)
RSV PCR (+)	10	26
RSV PCR (-)	95	606
	105	632

Supplementary Table S4:
Sensitivity analyses for PAF calculations, including deaths that remained ‘uncertain’ after adjudication either as Respiratory vs. non-Respiratory deaths.

Deaths by location	PAF<br>Cases adjudicated as ‘uncertain’ added to ALRI ‘Yes’<br>% (95% CI)	PAF<br>Cases adjudicated as ‘uncertain’ added to ALRI ‘No’<br>% (95% CI)
All deaths combined	2.8 (1.0-4.6)	2.4 (0.7-4.2)
Community deaths	3.9 (1.2-6.5)	3.6 (1.1-6.1)
Late facility deaths (occurring >/= 48 hours from admission)	1.2 (0-3.6)	0.8 (0-3.1)

Supplementary Figure S1: Distribution of cycle threshold values among infants where RSV was detected on PCR

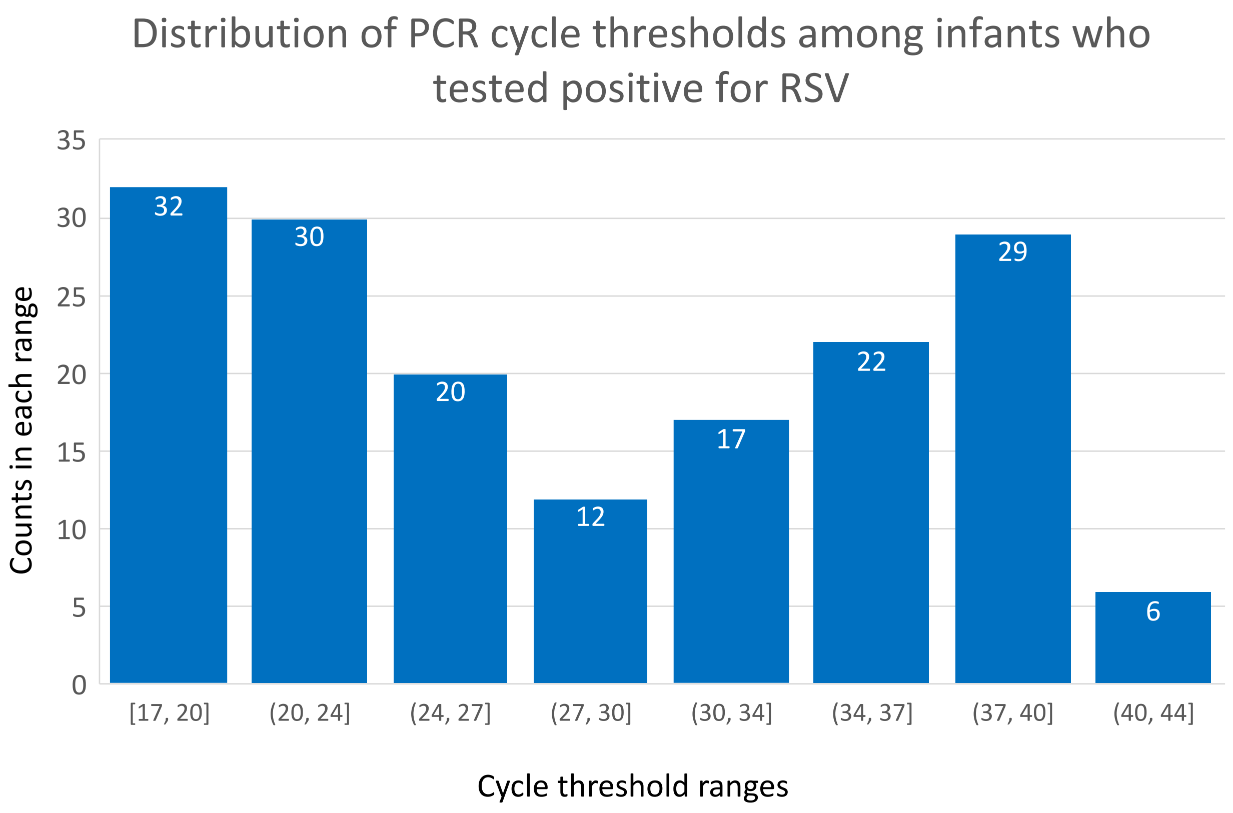

The figure summarizes the proportion of detecting PCR assays occurring within different strata of cycle threshold values. PCR was run for 45 cycles, making this the effective limit of detection. The median Ct value was 27.8 and 96% of all positive results fell below the a priori threshold of a Ct<40. An additional six infants had a detectable RSV signal above a Ct of 40 and could be further examples of RSV deaths but were not included in our analyses.

Supplementary Figure S2. Distribution of RSV deaths by Lusaka city districts and wards

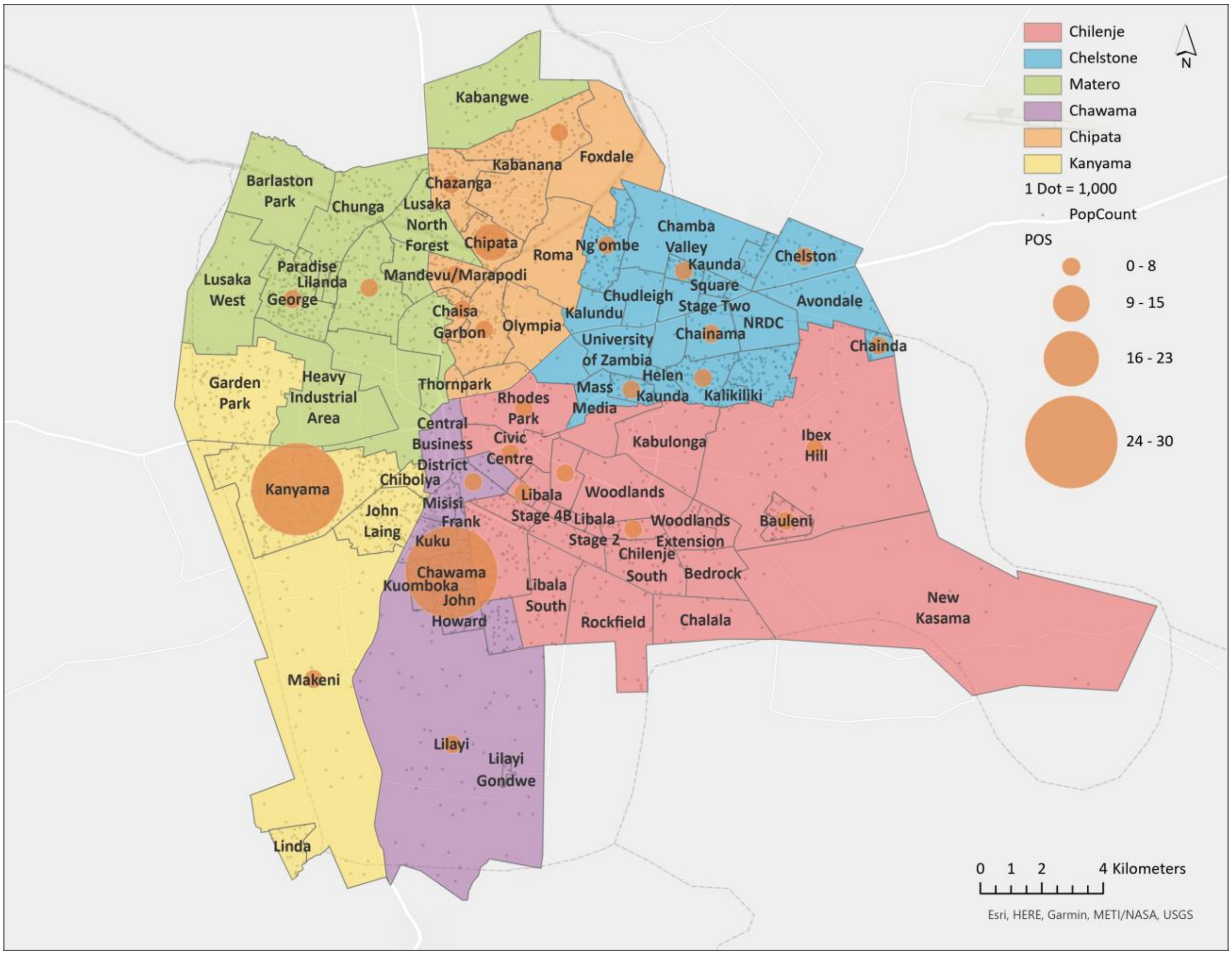

We used ArcGIS (Esri Inc, Redlands, CA) software to map the locations of the deaths within Lusaka. We pulled population size data from the Zambia Data Hub: https://zambia-open-data-nsdi-mlnr.hub.arcgis.com/ , which is managed by the Government of Zambia through the Ministry of Lands and Natural Resources, and the Zambia Statistics Agency. The major sections of Lusaka are color coded. Population density is represented by the small dots across the city map, each representing 1000 persons. The number of RSV deaths are shown proportionally and by color intensity in the overlying circles. RSV deaths were most prevalent in the three poorest and most densely-populated townships of Kanyama, Chipata and Chawama; and least prevalent in the more affluent sections of Lusaka, Matero, Chilenje and Chelstone.

Supplementary Figure S3. Duration of hospitalization prior to an RSV(+) death among infants

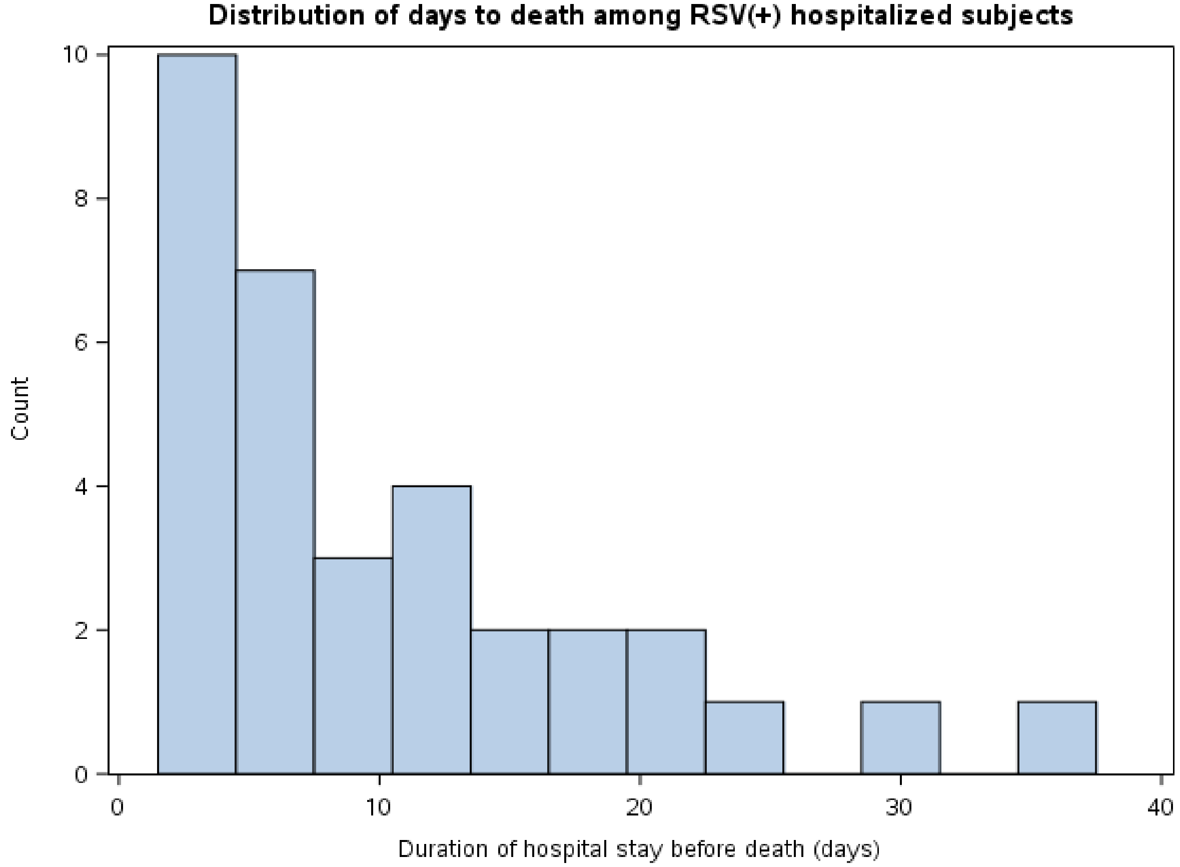

The figure summarizes data for infants with duration of hospitalization data, all of whom were attended at University Teaching Hospital Lusaka. Here we focus on late hospital deaths, i.e., those occurring >/= 48 hours after hospitalization. This accounts for the absence of deaths on the far left of the figure, as these were within that first 48 hours. These data presumably are a mixture of RSV deaths of community origin that occurred after 48 hours, plus, as one moves further and further to the right, an increasing proportion of nosocomial origin. Within this set of 46 deaths were 12 infants who were born at UTH and did not survive to discharge, who represent nosocomial infections by definition. Typically RSV has a 4-6 day incubation period, though viral shedding may last for a week or more. This makes it difficult or impossible to clearly define a point in time when cases are nosocomial. However, given the incubation period, it is increasingly likely that cases past day 5 are nosocomial. From this, we can draw some boundaries around the nosocomial fraction, ranging from 33% (12/36) to 64% (23/36) of late hospital RSV deaths.
